# Sublethal doses of insecticide reduce thermal tolerance of a stingless bee and are not avoided in a resource choice test

**DOI:** 10.1098/rsos.230949

**Published:** 2023-11-22

**Authors:** Holly Farnan, Peter Yeeles, Lori Lach

**Affiliations:** College of Science and Engineering, James Cook University, PO Box 6811, Cairns, Queensland 4870, Australia

**Keywords:** Apidae, heat stress, imidacloprid, fipronil, *Tetragonula hockingsi*, critical thermal maximum

## Abstract

Insecticides and climate change are among the multiple stressors that bees face, but little is known about their synergistic effects, especially for non-*Apis* bee species. In laboratory experiments, we tested whether the stingless bee *Tetragonula hockingsi* avoids insecticide in sucrose solutions and how *T. hockingsi* responds to insecticide and heat stress combined. We found that *T. hockingsi* neither preferred nor avoided sucrose solutions with either low (2.5 × 10^−4^ ng µl^−1^ imidacloprid or 1.0 × 10^−4^ ng µl^−1^ fipronil) or high (2.5 × 10^−3^ ng µl^−1^ imidacloprid or 1.0 × 10^−3^ ng µl^−1^ fipronil) insecticide concentrations when offered alongside sucrose without insecticide. In our combined stress experiment, the smallest dose of imidacloprid (7.5 × 10^−4^ ng) did not significantly affect thermal tolerance (CT_max_). However, CT_max_ significantly reduced by 0.8°C (±0.16 SE) and by 0.5°C (±0.16 SE) when bees were fed as little as 7.5 × 10^−3^ ng of imidacloprid or 3.0 × 10^−4^ ng of fipronil, respectively, and as much as 1.5°C (±0.16 SE) and 1.2°C (±0.16 SE) when bees were fed 7.5 × 10^−2^ ng of imidacloprid or 3.0 × 10^−2^ ng of fipronil, respectively. Predictions of temperature increase, and increased insecticide use in the tropics suggest that *T. hockingsi* will be at increased risk of the effects of both stressors in the future.

## 1. Introduction

Bees are critical components of natural and agricultural ecosystems [1], and concern is growing about declines in their populations [2–4]. These declines are likely driven by myriad stressors including habitat loss, pathogens and parasites, competition from introduced species, poor nutrition and insecticide exposure [1,2]. The effects of these stressors will likely be exacerbated by global climate change [3,5–7].

Insecticides have been blamed for bee deaths across the globe [8–10]. Bees are exposed to insecticides in the nectar and pollen of treated plants [11] and exposure is expected to become more prolific with increased insecticide use under agricultural intensification [12,13]. Imidacloprid is a neonicotinoid, which is the most widely used class of insecticides worldwide. Of the insecticide classes, neonicotinoids are the most often implicated in bee declines [3,4]. Neonicotinoids are neurotoxins derived from the natural compound nicotine and cause overstimulation, paralysis and death [14]. Sublethal effects, such as difficulty in learning and decreased foraging and homing ability, have also been observed in *Apis mellifera* and *Bombus terrestris* [15–17]. Fipronil is another widely used insecticide with lethal and sublethal effects on insect pollinators [18]. Fipronil is part of the phenylpyrazole chemical family and is an entirely synthetic insecticide that inhibits γ-aminobutyric acid (GABA) receptors in insects, leading to excess neural activity causing insects to experience muscle and nerve hyperexcitability, paralysis, and eventually death [19,20]. Fipronil also has sublethal effects on bees, for example reduced motor activity in *A. mellifera* [21], reduced climbing speed in the stingless bee *Melipona scutellaris* [22], and increased cell cytotoxicity of the mushroom bodies associated with memory in the stingless bee *Scaptotrigona postica* [23].

Lethal and sublethal effects of insecticides on bees could be reduced if bees avoided foraging on nectar and pollen contaminated with insecticides [24,25]; however, evidence that bees avoid consuming insecticide-contaminated resources is limited and conflicting. Individual *B. terrestris* workers given sucrose treated with the neonicotinoid imidacloprid (10 µg kg^−1^ and 100 µg kg^−1^) subsequently reduced their consumption of resources containing imidacloprid over a four-day period [26]. Individual *Bombus impatiens* workers exposed to both field relevant doses of imidacloprid in sucrose and control sucrose did not prefer sucrose containing imidacloprid (0.25 µg kg^−1^, 1 µg kg^−1^, 5 µg kg^−1^ and 10 µg kg^−1^) across a series of time points [27]. Avoidance of neonicotinoids has also been demonstrated by pollinating beetles and flies offered a choice between a sublethal concentration of imidacloprid (1.0 µg l^−1^, 0.1 µg l^−1^ and 0.01 µg l^−1^) and a control in pan traps [28] and by gravid mosquitoes that failed to lay eggs in water with either imidacloprid or chlorpyrifos [29]. In contrast, *A. mellifera* and *B. terrestris* given a choice between a sucrose solution and a sucrose solution with a sublethal dose of either of the neonicotinoids imidacloprid, thiamethoxam or clothianidin (1 nM, 10 nM, 100 nM and 1000 nM) consumed more sucrose solution with either imidacloprid or thiamethoxam than sucrose alone, possibly because of the pharmacological action of these compounds on nicotinic acetylcholine receptors in the bees' brains [30]. *Bombus terrestris audax* increased visits to insecticide-laced sucrose feeders, indicating a preference for thiamethoxam at 2 µg kg^−1^ and 11 µg kg^−1^ over untreated sucrose [31]. Studies that test whether other groups of social bees, such as stingless bees (Meliponini), prefer or avoid neonicotinoids are limited. The stingless bee *Nanotrigona perilampoides* consumed more sucrose containing imidacloprid (LC_20_—a lethal concentration that kills 20% of test subjects) and insecticide free sucrose than sucrose containing imidacloprid (LC_10_—a lethal concentration that kills 10% of the test subjects) [32]. The authors attribute their results to bees exposed to LC_10_ attempting to consume as little sugar as possible as a behavioural defence mechanism to avoid imidacloprid intoxication, whereas bees exposed to LC_20_ may have experienced physiological stress, and sucrose consumption may have been necessary for them to meet energy requirements for metabolic pathways and detoxifying capabilities [32]. In contrast*,* the stingless bee *Tetragonula laeviceps* chose honey over both honey containing the insecticides alpha-cypermethrin and spinetoram and the insecticides alone in a Y-tube olfactometer dual choice aroma assay [33]. Given the equivocal results and paucity of studies, further research into insecticide preference/avoidance in stingless bees in particular is warranted.

Heat stress associated with climate change-driven extreme heat events is another major stressor that creates challenges for bees, including impacts on foraging activity, pollination services, task-related physiology, immunocompetence, reproductive capacity, growth and development of bees [34–37]. For example, heat stress damages the fertility of queen bees and the digestive tracts of worker bees [38,39] and can trigger malformations of the proboscis, stinger, wings and legs of *Apis mellifera carnica* [40]*.* Further, bee communities and their composition are shifting with climate change [41,42]. These shifts may be arising due to mismatches between environmental temperatures and organisms’ physiological tolerances [43,44].

Insecticide toxicity and heat stress may have interactive effects, but to date they have not been well studied in many insects including bees. Within the small number of studies investigating these interactive effects, the results are equivocal. Heat stress and several insecticides, including imidacloprid and fipronil, act on the nervous system [45–52]. There is evidence from *A. mellifera* that the combined effect of insecticide exposure and heat stress could result in higher heat tolerance or synergize and cause higher mortality. For example, acute oral exposure to the neonicotinoids imidacloprid and acetamiprid increased thermal tolerance of *A. mellifera* by as much as 4.3°C [53]. Conversely, *A. mellifera* fed three concentrations (0 ppb, 5 ppb and 20 ppb) of imidacloprid and maintained at temperatures (26°C (below optimal) and 38°C (above optimal)) were more susceptible to imidacloprid, with significantly higher mortality compared to the control (32°C) and showed altered gene regulation [54]. Another study demonstrated that *A. mellifera* colonies treated with imidacloprid (20 ppb for 14 days) and high temperature (41°C for 6 h) exhibited altered metabolic pathways [55]. One of the principal mechanisms used by insects to escape adverse effects of both natural and synthetic toxins, such as nicotine and the neonicotinoids, is metabolic resistance [51]. However, *A. mellifera* have fewer numbers of detoxifying genes than other insects, leaving them more sensitive to some insecticides [56]. Further, it is metabolically and energetically costly for bees to detoxify toxins [51]; this could lead to other impacts on the bees' health. With predicted increases in temperatures and insecticide use globally [57–59], research that further elucidates the effect of these combined stressors is essential.

While a growing body of work is revealing the effects of stressors on *A. mellifera*, we know relatively little about how the thousands of other bee species respond to environmental stressors. Stingless bees are the most diverse group of eusocial bees with over 500 described species distributed throughout the tropical and subtropical regions of Africa, Asia, Oceania, and the Americas [60,61]. They provide pollination services to a wide diversity of tropical plants and crops [60,62]. Several stingless bee species have increased sensitivity to insecticide toxicity compared to *A. mellifera* due to differences in body size, physiology, behaviour, and metabolism [23,63,64], and laboratory tests have shown that neonicotinoids are among the most toxic compounds to stingless bees [65–67]. Most research focus has been on the genera *Melipona*, *Scaptotrigona* and *Nannotrigona*, with effects on the 55 other melipone genera little studied [63]. Furthermore, as tropical species, stingless bees are likely to tolerate a narrower range of temperatures and may be living close to their upper thermal limits compared to temperate species [68]. This may result in stingless bees being more susceptible to the effects of climate change related heat stress.

**Figure 1.**
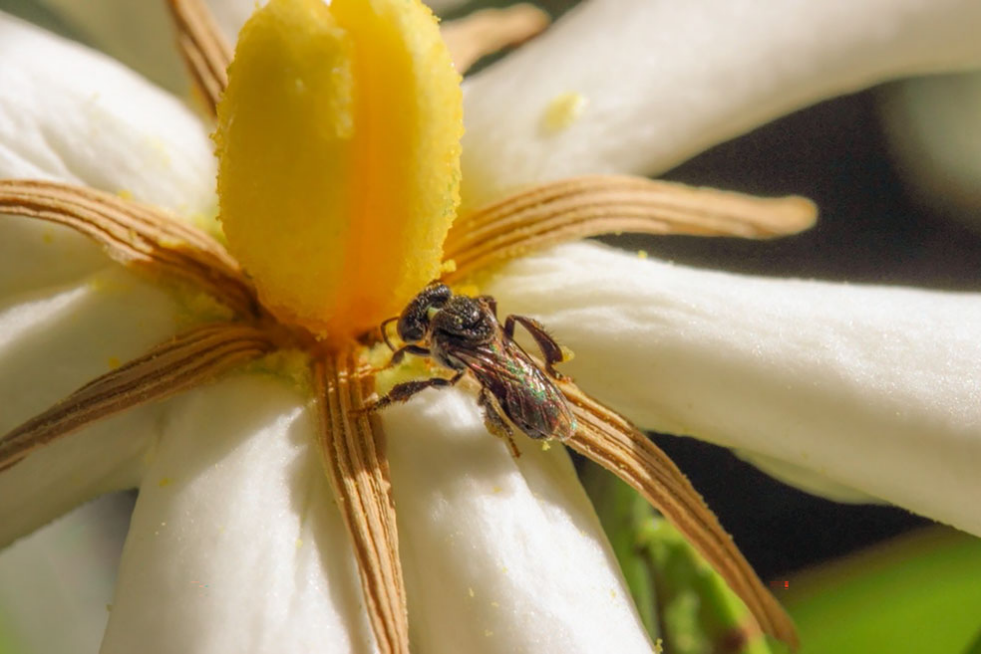
*Tetragonula hockingsi* collecting pollen from flower. Photo credit: Campbell Simpson, 2021.

We assessed how the stingless bee *Tetragonula hockingsi* responds to insecticide laced sucrose (mimicking floral nectar) and then investigated how *T. hockingsi* responds to the combined stress of insecticide exposure and heat stress*.* We asked whether *T. hockingsi* avoided, preferred, or remained indifferent to both high and low sublethal concentrations of either the neonicotinoid imidacloprid or fipronil when given the choice between sucrose solutions with and without one of these insecticides. We then investigated the effect of three different sublethal doses of these insecticides on thermal tolerance of *T. hockingsi* (figure 1).

## 2. Material and methods

### 2.1. Study organism

*T. hockingsi* are distributed widely across the Australian tropics and provide pollination services for many native plants and crops such as mango, avocado, and macadamia [60]. *T. hockingsi* is one of the two species of stingless bees commonly used for commercial crop pollination in Australia [62]. They possess common stingless bee traits such as being active year-round and nesting in various cavities including hollow trees, buildings and rock crevices [60,69]. On average, there are approximately 10 000 workers in each nest [70]. Nests are insulated by an involucrum (an enveloping membrane to protect and insulate the brood composed of propolis, cerumen and resins) [69] and *T. hockingsi* can modify nest temperatures by active ventilation at the entrances. This involves workers fanning their wings while facing outward towards the entrance to draw cool air into the nest [69].

### 2.2. Field collection and laboratory conditions

**Figure 2.**
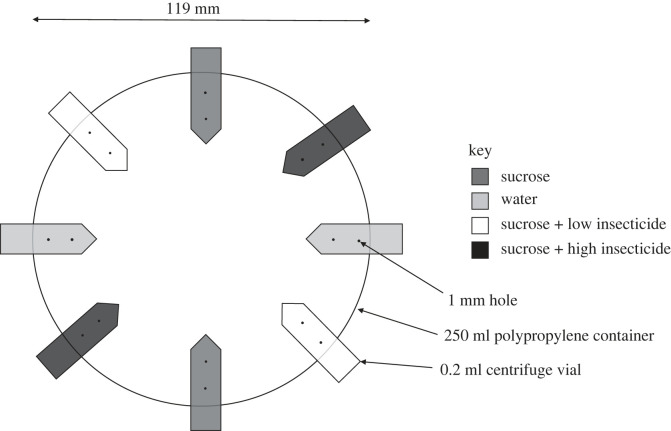
Diagram of experimental arena (240 ml, 119 mm diameter × 38 mm height) fitted with 0.2 ml Eppendorf vials with different shades indicating treatment types.

For the choice tests, we collected adult *T. hockingsi* from one hive, located at the Cairns Botanic Gardens (16° 53′ 57.882″ S, 145° 44′ 49.998″ E) and two hives at Kewarra Beach (16° 78′ 43.58″ S, 145° 68′ 85″ E) in northern Queensland, Australia in June and July of 2021. Bees were collected fresh on the day of each assay between 08.00 and 08.30 in which ambient temperatures were between 21°C and 24°C. Hives were selected based on confirmed identification of *T. hockingsi* via genetic analysis (primers described in [71]) to amplify a 299 bp fragment of the mitochondrial gene cytochrome oxidase I (mt-COI) in *T. hockingsi* (sequences: *Barhock_R:* AAGGCCGAATCCTGGAAGAA and *T_hock_COI_spec_F5:* GAATTTCATCTATTCTTGGA) by Dr Ros Gloag, The University of Sydney. We collected exiting foragers by placing our experimental arenas (240 ml, 119 mm diameter × 38 mm height polypropylene containers) over the hive entrance. We fitted arenas with a mesh window (approx. 60 mm × 20 mm) that could be opened and closed to add or remove foragers as necessary to achieve 12 bees per arena. Piloting revealed 12 bees per arena was sufficiently high so that bees would not huddle together and low enough to enable all bees to have access to the solutions during the experiment. Arenas were fitted with eight 0.2 ml Eppendorf vials, each with two 1 mm holes for feeding, based on the design used by Kessler *et al*. [30]. Vials were evenly spaced around the wall of the arenas and inserted horizontally (figure 2). Empty feeding tubes were fitted during field collection to prevent bees from escaping from holes. The use of a single arena for field collection and the choice tests reduced handling of bees. Bees in each arena were initially provided with a piece of cotton wool soaked in 2 ml of 25% (w/v) sucrose solution immediately after capture. Piloting revealed that this concentration was sufficiently attractive to *T. hockingsi* and is within the range of sugar concentration in nectar [72]. Bees were then transported inside arenas covered with a loose dark cloth to a temperature- and humidity-controlled chamber (WiseCube model TEMI850) set at 27°C and 50% relative humidity whereby they remained for an acclimatization period of 3 h. After this period, sucrose-soaked cotton was removed, and experimental treatments were applied between 11.00 and 11.30 (depending on when they were collected). After 24 h the experiments were ended, and data were collected.

For the thermal tolerance experiment, we collected adult *T. hockingsi* throughout July and August of 2021 from the same Cairns Botanic Gardens hive as for the choice tests. Using the same method and container design as detailed above, we captured approximately 40 exiting foragers on each collection day for eight days from which we arbitrarily selected 24 for that day's experimental replicates (temperature ramp bees *n* = 12, temperature control bees *n* = 12). After the eight days, temperature ramped bees *n* = 85 (12 bees × 6 insecticide treatments + 13 control bees (1 × extra bee ran on day 6)) and temperature control bees *n* = 84 (12 bees × 6 insecticide treatments + 12 control bees). We captured more bees than required to allow for replacement in case of death due to handling effects; however, in all cases extra bees were not needed as there were no mortalities prior to experiments. Collections occurred 24 h prior to each experiment, between 08.00 and 08.30. Following capture, bees were maintained under the same conditions as detailed above for an acclimation period of 24 h. We removed the sucrose-soaked cotton between 08.00 and 08.30 the next day to fast the bees for 1 h prior to running the experiment.

### 2.3. Selection of insecticides, concentrations and exposure route

For both the choice tests and determination of thermal tolerance, we separately tested two insecticides: the neonicotinoid imidacloprid (Pestanal, Sigma-Aldrich) and fipronil (Termidor Residual 100 g fipronil/L, BASF Australia Ltd), a synthetic neurotoxin. We chose these insecticides because they are widely used, have been implicated in mass mortalities of bees, can be systemic, and bees are easily exposed to them in the nectar and pollen of treated crops [73,74]. For the choice tests, we tested low (2.5 × 10^−4^ ng µl^−1^ and 1.0 × 10^−4^ ng µl^−1^) and high (2.5 × 10^−3^ ng µl^−1^ and 1.0 × 10^−3^ ng µl^−1^) concentrations of imidacloprid and fipronil, respectively. For the thermal tolerance experiment, we tested the same low and high concentrations of each insecticide and added a third very high (2.5 × 10^−2^ ng µl^−1^ and 1.0 × 10^−2^ ng µl^−1^) concentration for imidacloprid and fipronil, respectively (hereafter referred to as low, high and very high for each insecticide). When selecting insecticide concentrations, we evaluated three approaches. Firstly, we considered basing concentrations from amounts of residues found in nectar and pollen; however, there were no data available for Australia. We then considered basing concentrations on known toxicities for the species; however, at the time of the experiments there were no published toxicities for any *Tetragonula* species for either insecticide. Finally, we decided to use studies of other bee species and scaled concentrations to body size [23,33,75] (electronic supplementary material, appendix 1). We chose oral exposure over contact exposure because oral exposure is the most likely route of exposure for bees [76]. Bees commonly consume insecticide residues that have been applied as seed treatments, soil treatments, and foliar sprays. Insecticide residues may be expressed in plant nectar and pollen, guttation fluid, and honeydew [77].

We prepared fresh 200 ml of 25% (w/v) sucrose solution daily of which we set aside 50 ml as our control sucrose solution. We then added either 1278 ng of imidacloprid or 500 ng of Termidor (fipronil) to separate beakers containing 50 ml of sucrose solution, to obtain the solutions with the highest concentrations we tested. We then diluted these solutions to obtain the lower concentrations. We vortexed each treatment for 20 s (WiseMix model VM-10 vortex) and then immediately pipetted 0.2 ml of the solution into its corresponding vial and gently tapped each vial to ensure that no air bubbles were present. Each arena contained two vials of each treatment to allow for more opportunity for bees to feed. All vials were arranged in an alternating pattern so that no two of the same treatments were next to each other and the same arrangement of vials was set for each arena. Two vials of water were added to each arena in case bees required water for hydration or thermoregulation [30]. Vials containing water were not included in data analysis. We calculated the amount of solution consumed from each tube as the difference in the mass of each vial after 24 h using a microbalance (A&D model HR-200, accuracy ±0.01 mg) minus the average evaporation control for the respective treatment [30]. For each insecticide, we repeated the experiment with 30 cohorts (10 from each hive) of 12 bees each. Each trial was conducted on a fresh cohort of bees with the same arrangement of vials. After each experiment, we held all bees for an additional 72 h to confirm that our concentrations were truly sublethal. All 720 bees survived this period.

### 2.4. Determination of heat tolerance

To assess thermal tolerance, we used the measure of critical thermal maxima (CT_max_, a measure of the highest temperature at which an organism can maintain neuromuscular function [78]). Following acclimation, we transferred temperature ramp bees and control bees to individual 2 ml Eppendorf vials with three 1 mm air holes. We piloted different sized vials and found that this vial size minimized stress and allowed bees to avoid incidental contact with the treatment solution. We determined the mass of each individual bee by pre-weighing vials using a microbalance (A&D model HR-200, readability(mg): 0.1) and then reweighing once the bee was inside.

After a 1 h fasting period. Each bee was given 3 µl of either 25% (w/v) sucrose solution or a low, high, or very high concentration of imidacloprid (2.5 × 10^−4^ ng µl^−1^, 2.5 × 10^−3^ ng µl^−1^ and 2.5 × 10^−2^ ng µl^−1^, respectively) or fipronil (1.0 × 10^−4^ ng µl^−1^, 1.0 × 10^−3^ ng µl^−1^ and 1.0 × 10^−2^ ng µl^−1^, respectively) in 25% (w/v) sucrose. The 3 µl volume given at these concentrations yielded doses of 7.5 × 10^−4^ ng, 7.5 × 10^−3^ ng and 7.5 × 10^−2^ ng of imidacloprid and 3.0 × 10^−4^ ng, 3.0 × 10^−3^ ng and 3.0 × 10^−2^ ng of fipronil. Bees were assigned to treatments randomly, and the observer (H.F. for all trials) was kept blind to treatment throughout the experiment. We prepared all solutions fresh daily and agitated them for 20 s using a vortex (WiseMix model VM-10) prior to administering to the bees. Each treatment was applied to four bees each day (= 28 bees total across the seven solutions) with half of the bees temperature ramped and half controlling for the ramping (kept at 26°C in a temperature-controlled cabinet). Five bees did not completely consume the solution after 6 h and were excluded from the experiment. An additional five bees were run on another day to achieve 12 bees for each treatment.

We determined the CT_max_ of *T. hockingsi* by ramping a temperature increase from an ambient temperature at a set rate [79]. Each day, we transferred the bees to be temperature ramped to individual 5 ml vials sealed with parafilm to prevent water leakage while submerged. Vials were placed into randomized positions in a vial rack positioned in a water bath made by attaching a temperature-controlled immersion heater (Westinghouse WHSV01K) to a 9 l rectangular plastic tub, following a similar design used by Nacko *et al*. [80] to measure the CT_max_ of *T. hockingsi* [80] (electronic supplementary material, appendix 2). The temperature-controlled immersion heater included a jet that kept water well mixed, and we confirmed this by using two thermocouple probes at opposite sides of the bath (at depths in line with the highest and lowest vials) to check that temperature was homogeneous throughout the water bath. We stabilized the water bath temperature for 15 min at 26°C prior to the experiment. The water temperature was calibrated against a HOBO temperature logger (model MX2202, accuracy ±0.5°C) that was placed in the middle of the tub. We ramped bees from an acclimation temperature of 26.2 (± 0.2 SD)°C by increments of 0.5°C every 2 min until the CT_max_ was reached for all individuals. We identified CT_max_ as the temperature at which an individual became unresponsive to a stimulus [81,82], which was in this case a single flick of the vial (vial was removed from water bath to administer stimulus and replaced if bee responded [81,83,84]). During the temperature ramp, bees exhibited typical indicators of heat stress, initially including wing fluttering, or extending and holding still a single or both wings [82]. As bees approached their CT_max_, they were unable to right themselves and lost muscular coordination and began spasming. Spasms were often fast and whole-bodied initially, before becoming slower uncontrolled movements of the limbs. Bees eventually became still with head and abdomen adducted, however continued to respond to stimulus through the movement of antennae and limbs. We checked for responses at every 2°C increase until 38°C, if necessary (i.e. we only provided stimulus if individuals were motionless), then at 0.5°C increments beyond this.

We held the temperature ramp control bees (*n* = 14 for each day) at the constant temperature of 26°C in a temperature-controlled cabinet, to assess whether mortality would occur due to insecticide treatment only, over the same period that the temperature ramp experiment took place. These bees had received the same insecticide and sucrose control doses as the bees that were temperature ramped. To ensure that temperature control bees experienced the same handling effects as their temperature-ramped counterparts, they were also transferred into individual 5 ml vials sealed with parafilm and stratified in a vial rack prior to placement in the temperature-controlled cabinet.

### 2.5. Statistical analysis

We conducted statistical analysis in R v. 1.4.1103 [85]. For the choice tests, we used a generalized linear model (GLM) [85] with a quasibinomial error distribution for each insecticide to compare the amount of each sucrose solution consumed (the combined value of two vials of the same treatment for each arena) expressed as a proportion of the total amount consumed from the six vials of solutions (i.e. excluding water) in each arena (hereafter referred to as relative proportion). Treatment (i.e. sucrose, water, low concentration of insecticide and high concentration of insecticide), hive, and the interaction between treatment and hive were fixed effects. We tested each model against a null model using a type 3 ANOVA from the ‘car’ R package [86]. We conducted *post* *hoc* Tukey HSD tests (emmeans v. 1.5.2-1) [87] to determine whether there were significant differences across treatments and hives.

We used a linear mixed model with a Gaussian error distribution for each insecticide to test whether insecticide exposure influenced CT_max_ for *T. hockingsi* workers, with CT_max_ as the response variable and insecticide treatment and bee mass as fixed effects (LMM; lme4 v. 1.1-25) [88]. We initially included an interaction between insecticide dose and bee mass and dropped it from the model when they did not improve model fit by more than 2 ΔAIC [89]. To account for variation among days, we included ‘day of assay’ as a random effect. We conducted *post hoc* Tukey HSD tests (emmeans v. 1.5.2-1) [87] to determine whether there were significant differences in CT_max_ across sucrose-control and insecticide treatments. Analysis was conducted on temperature-ramped bees only. No analysis was needed for the temperature control bees as they had a 100% survival rate.

We tested all models to ensure they met assumptions of homogeneity of variance, and independence and normality of residuals by plotting and visually inspecting our data using the ‘qqnorm’ functions in R [85] against the error distributions.

## 3. Results

### 3.1. Choice tests

**Figure 3.**
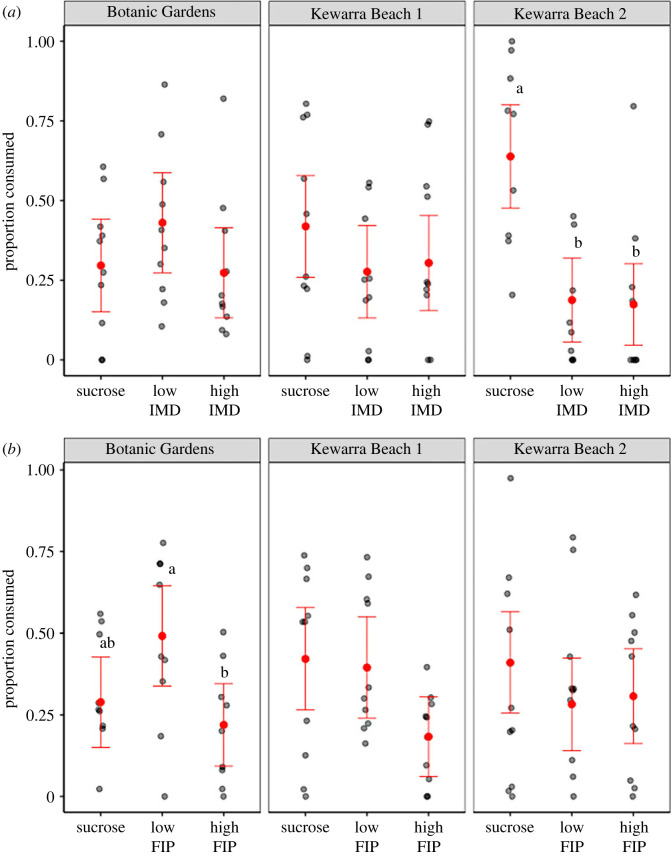
Mean (±95% CI) amount of each treatment consumed as a proportion of the total amount of sucrose-based solutions consumed in each arena, across hives for (*a*) imidacloprid concentrations (= IMD, low = 2.5 × 10^−4^ ng µl^−1^, high = 2.5 × 10^−3^ ng µl^−1^) and (*b*) fipronil concentrations (= FIP, low = 1.0 × 10^−4^ ng µl^−1^, high = 1.0 × 10^−3^ ng µl^−1^). Error bars without a letter in common above denote significant differences among treatments (Tukey HSD; *p* < 0.005).

For the imidacloprid choice tests, the relative proportion consumed did not vary significantly among treatments, but there was a significant variation by hive and a significant interaction between treatment and hive (table 1), reflecting that hives responded to treatments differently. *Post* *hoc* comparisons among solutions within hives revealed that the relative proportion of each treatment consumed did not differ significantly among treatments for bees from the Botanic Gardens hive or Kewarra Beach hive 1. However, bees from Kewarra Beach hive 2 consumed a significantly higher relative proportion of insecticide-free sucrose than sucrose containing a low concentration of imidacloprid (Tukey HSD; *p* = 0.001) and sucrose containing a high concentration of imidacloprid (Tukey HSD; *p =* 0.0007) (figure 3*a*).

**Table 1.** Summary of statistical analyses for both experiments showing ANOVA output of generalized linear models for imidacloprid and fipronil choice tests and linear mixed models for comparison of CT_max_. Values of *p* less than 0.05 are in bold.

	insecticide		Chisq	d.f.	*p* value
choice tests	imidacloprid	treatment	2.4423	2	0.29
		hive	8.7794	2	**0.0124**
		treatment:hive	15.7919	4	**0.0033**
	fipronil	treatment	7.2931	2	**0.0261**
		hive	1.8829	2	0.39
		treatment:hive	7.2948	4	0.12
comparison of CT_max_	imidacloprid	(intercept)	10493.3187	1	<**0.001**
		treatment	127.8725	3	<**0.001**
		bee mass	0.1977	1	0.66
	fipronil	(intercept)	13663.1184	1	<**0.001**
		treatment	69.7993	3	<**0.001**
		bee mass	0.6383	1	0.42

For the fipronil choice tests, the relative proportion consumed varied significantly among treatments, but not by hive (table 1). There was no significant hive by treatment interaction (table 1). *Post* *hoc* comparisons among treatments within hives revealed that *T. hockingsi* from the Botanic Gardens hive consumed a significantly higher relative proportion of sucrose with a low concentration of fipronil than sucrose with a high concentration of fipronil (Tukey HSD; *p* = 0.0315; figure 3*b*). The relative proportion of insecticide-free sucrose consumed did not differ significantly from solutions with either low or high fipronil concentrations for any of the hives (figure 3*b*).

The findings from our choice experiments suggest that *T. hockingsi* do not avoid sublethal concentrations of imidacloprid or fipronil, possibly due to an inability to detect such substances or due to indifference, either of which suggest that *T. hockingsi* will not actively avoid exposure to these insecticides in nectar.

### 3.2. Comparison of thermal tolerance

**Figure 4.**
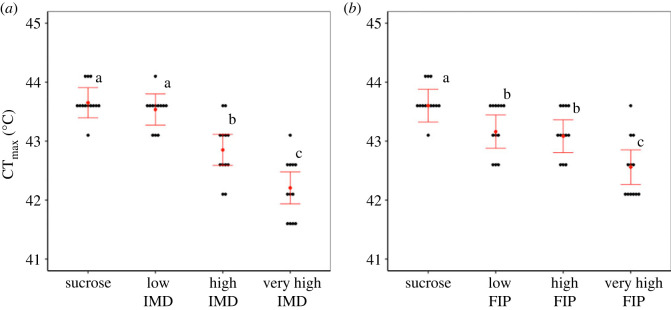
Mean (±95% CI) CT_max_ for *Tetragonula hockingsi* workers (from Cairns Botanic Gardens hive) given (*a*) sucrose solution and low (7.5 × 10^−4^ ng), high (7.5 × 10^−3^ ng) and very high (7.5 × 10^−2^ ng) doses of imidacloprid in sucrose (IMD, *n* = 12 for all treatment groups), and (*b*) sucrose control solution and low (3.0 × 10^−4^ ng), high (3.0 × 10^−3^ ng) and very high (3.0 × 10^−2^ ng) doses of fipronil (FIP, *n* = 12 for all treatment groups). Points indicate individual bees. Error bars in the same panel without a letter in common above them significantly differ (*p* < 0.05).

Consumption of imidacloprid and fipronil each significantly decreased the thermal tolerance of *T. hockingsi* individuals (figure 4, table 1). For imidacloprid, *post* *hoc* pairwise comparisons revealed a significantly lower mean CT_max_ in bees that received the high and very high doses compared to bees that received sucrose only (Tukey HSD; *p* < 0.0001 for both comparisons) and bees that received the low dose (Tukey HSD; *p* = 0.0002, *p* < 0.0001, respectively) (figure 4*a*). CT_max_ was also lower in bees that received a very high dose compared to bees that received a high dose (Tukey HSD; *p* = 0.0004) (figure 4*a*). CT_max_ did not differ between bees that received sucrose only and bees that received a low dose of imidacloprid (figure 4*a*).

For fipronil, *post* *hoc* pairwise comparisons revealed a significantly lower mean CT_max_ between the sucrose control bees and those that received low (Tukey HSD; *p* = 0.0022), high (Tukey HSD; *p* = 0.0003) and very high doses (Tukey HSD; *p* < 0.0001) (figure 4*b*). *Post* *hoc* comparisons also revealed significantly lower mean CT_max_ values between bees that received low and very high doses (Tukey HSD; *p* = 0.0004) and bees that received high and very high doses (Tukey HSD; *p* = 0.0008) (figure 4*b*). CT_max_ did not differ between bees that received low and high doses of fipronil (figure 4*b*).

Mean bee mass was 8.4 (± 10 SD) mg. Mass did not have a significant effect on CT_max_ of *T. hockingsi* individuals exposed to either insecticide (table 1).

All 84 bees that were exposed to the seven insecticide treatments but not the temperature ramp survived for the duration of the temperature ramping experiment conducted the same day (approx. 120 min).

## 4. Discussion

Insecticide exposure and climate change are widely acknowledged as key threats to bees, yet there has been little work investigating their interactive and potentially synergistic effects, especially for non-*Apis* bee species. Furthermore, little is known about whether bees can avoid these stressors. Our experiments revealed no consistent avoidance of either imidacloprid- or fipronil-laced sucrose solutions by *T. hockingsi*, as bees from only one out of three hives avoided imidacloprid and none avoided fipronil. We also found that the CT_max_ (hereafter termed thermal tolerance) of individuals was significantly reduced by 0.8°C (±0.16 SE) and by 0.5°C (±0.16 SE) when bees were fed as little as 7.5 × 10^−3^ ng of imidacloprid or 3.0 × 10^−4^ ng of fipronil, respectively, and as much as 1.5°C (±0.16 SE) and 1.2°C (±0.16 SE) when bees were fed 7.5 × 10^−2^ ng of imidacloprid or 3.0 × 10^−2^ ng of fipronil, respectively. Our demonstration of diminished tolerance of heat stress at field relevant insecticide doses combined with predictions of increases in mean and extreme temperatures throughout the range of *T. hockingsi* [57,90] suggest that they will be at increased risk of the effects of both stressors in the future.

Stingless bee responses to insecticides broadly vary, with evidence of avoidance or repellence [32,33,91,92], exclusion of foragers exposed to insecticides [93], not avoiding individuals from their colony treated with insecticide [94] and not rejecting resources containing insecticides [95]. Demonstrations of stingless bee responses to imidacloprid are limited, and we are only aware of two other studies that tested a response of a stingless bee to imidacloprid. The first study demonstrated that *Nannotrigona aff. testaceicornis* antennated rather than avoided individuals from their colony that were treated topically with a sublethal dose of imidacloprid (3.5 × 10^−1^ ng per bee) [94]. The second study showed that *N. perilampoides* consumed more sucrose containing imidacloprid (LC_20_—a lethal concentration that kills 20% of test subjects) and insecticide free sucrose than sucrose containing imidacloprid (LC_10_—a lethal concentration that kills 10% of the test subjects) [32].

While we should not expect that all stingless bee species will respond to insecticides in the same way, the experimental designs in some of these studies provided more opportunity for the bees to detect insecticide via olfaction without gustation. Our study was designed more to test for responses to insecticide in nectar, and therefore a design that included a gustatory response was appropriate. Our findings raise concern given the high potential for stingless bees to forage upon flowering crops treated with insecticides in the field [62,76].

Our findings differ from previous work on other insects that found neonicotinoids to have an attractant [30,31], repellent [28], or antifeedant effect [26]. These differences in findings may be due to a concentration dependent effect, as neonicotinoids may act as an attractant in some concentrations but elicit a neutral response at others [30]. Differences in findings may also be due to the responses measured, experimental design, and insect physiology between stingless bees and other species. Differential responses to insecticides may also depend on the amount of previous exposure bees have experienced. For example, strong repellent effects of low concentrations of neonicotinoids in flies and beetles may be due to the widespread use of neonicotinoids in agricultural settings over previous years, leading to a strong selection for their avoidance [28]. Our results for *T. hockingsi* did not reveal strong repellent effects and align with the work of Muth *et al*. [27], where *B. impatiens* did not show a preference towards consuming either neonicotinoid-containing solutions or sucrose solutions.

Consumption of 3 µl of the same concentrations and insecticides that *T. hockingsi* failed to consistently avoid in our nectar choice experiments resulted in significantly lower thermal tolerance. The thermal tolerance of imidacloprid exposed bees declined by 0.8–1.5°C, and the thermal tolerance of fipronil exposed bees declined by 0.5–1.2°C. The survival of the temperature ramp control bees indicates that mortality in the temperature ramp experiment was due to the combination of insecticide exposure and thermal stress, and not just insecticide exposure alone. Our findings contrast with a recent study in which sublethal doses of imidacloprid increased thermal tolerance in *A. mellifera* [53]. The thermal tolerance of *A. mellifera* that received doses ranging from 0.18 ng to 3.6 ng of imidacloprid were on average 2.6°C to 4.3°C greater than the control group. The authors theorized that their results may be due to sublethal doses of imidacloprid activating a stress response in the bees, which in turn may facilitate greater heat resistance. The differences in results between our study and Gonzalez *et al*. [53] may be due to differences in body size and physiology between *T. hockingsi* and *A. mellifera*. For example, when comparing the sensitivity of bee species to 158 different pesticides, bees with greater mean weight (*B. terrestris* and *A. mellifera*) had lower sensitivity than smaller bees (*Nomia melanderi* and *Megachile rotundata*) [96]. Moreover, insect body mass often positively correlates with thermal tolerance [97–101], although not in all cases [102,103]. As *T. hockingsi* have a body mass approximately 1/15 that of *A. mellifera* [75], it is possible that *T. hockingsi* are more susceptible to the effects of insecticides and heat stress simply due to their smaller size [96]. Furthermore, responses to insecticide exposure and heat stress could be dictated at the gene level. For example, non-optimal ambient temperatures aggravate imidacloprid toxicity and affect *A. mellifera* gene regulation [54]. Further documentation of stingless bee biology will help us to elucidate their responses to the combined stress of insecticide exposure and heat stress in the future.

While the findings from both our choice assays and our thermal tolerance experiments provide important proxies for what may occur in the field, the responses of bees under field conditions may vary. For example, our choice assay only allowed for a 24 h exposure period to insecticides, whereas in the field, bees may develop preference for or avoidance of insecticide in nectar over longer time periods. CT_max_ is suggested to be a useful predictor of species' responses to climate warming in regions with relatively warm baseline temperatures where many species are already close to their upper thermal limits [104]. Given the tropical distribution of *T. hockingsi*, it is reasonable to assume that CT_max_ may be a reliable predictor of thermal tolerance for the species. However, there are some limitations of applying CT_max_ values directly to ambient temperatures. The temperature ramping protocols expose bees to a more rapid rise in temperature than is likely to occur under natural conditions [105]. Moreover, bees in a temperature ramping experiment cannot move to cooler microclimates or benefit from hive thermoregulation strategies such as fanning [106]. Another limitation of extrapolating ambient survival from CT_max_ values is that differences in thermal tolerance might occur among populations or among castes of social insects like bees [107]. Nevertheless, CT_max_ values provide an indicative measurement for what organisms may experience in the field under extreme thermal stress events. These measurements may help us to predict how the species will respond to changes in climate when also challenged by insecticide exposure.

Tropical rainforest temperatures have been increasing rapidly since the 1970s [57] and are predicted to continue, with global climate models projecting average temperature increases of 1–4°C at lower latitudes [90]. These projections suggest that global warming of less than 1°C will cause tropical regions to experience extreme conditions (i.e. temperatures exceeding 2 standard deviations from the mean) sooner than other regions of the globe [90]. Tropical areas in which *T. hockingsi* are distributed are already experiencing extreme heat waves. For example, the city of Cairns, which is well within the geographic range of this species, experienced an extreme heatwave in 2018 and recorded a high temperature of 42.6°C [108]. Furthermore, tropical species are expected to have low tolerance to increased temperatures given the relatively narrow range of temperatures they typically experience [109]. The tropical distribution of stingless bees puts them further at risk given the trends of increasing insecticide use in the tropics in particular, and the potential for combined effects of insecticide exposure and heat stress [10,96,110]. The results from this study provide evidence of how *T. hockingsi* may respond to the combined stress of changes in climate and insecticide exposure.

## Data Availability

Data are available at Research Data JCU (doi:10.25903/v799-7v58): https://research.jcu.edu.au/data/published/173e64c0342611ed907d2d60f024bc99. Electronic supplementary material is available online [111].
